# Loss of Mevalonate/Cholesterol Homeostasis in the Brain: A Focus on Autism Spectrum Disorder and Rett Syndrome

**DOI:** 10.3390/ijms20133317

**Published:** 2019-07-05

**Authors:** Marco Segatto, Claudia Tonini, Frank W. Pfrieger, Viviana Trezza, Valentina Pallottini

**Affiliations:** 1 Department of Biosciences and Territory, University of Molise, Contrada Fonte Lappone, 86090 Pesche (IS), Italy; 2 Department of Science, University Roma Tre, Viale Marconi, 446, 00146 Rome, Italy; 3 Institute of Cellular and Integrative Neurosciences (INCI) CNRS UPR 3212, Université de Strasbourg, 5, rue Blaise Pascal, 67084 Strasbourg Cedex, France

**Keywords:** autism spectrum disorder, brain, cholesterol, mevalonate pathway, Rett syndrome

## Abstract

The mevalonate (MVA)/cholesterol pathway is crucial for central nervous system (CNS) development and function and consequently, any dysfunction of this fundamental metabolic pathway is likely to provoke pathologic changes in the brain. Mutations in genes directly involved in MVA/cholesterol metabolism cause a range of diseases, many of which present neurologic and psychiatric symptoms. This raises the question whether other diseases presenting similar symptoms are related albeit indirectly to the MVA/cholesterol pathway. Here, we summarized the current literature suggesting links between MVA/cholesterol dysregulation and specific diseases, namely autism spectrum disorder and Rett syndrome.

## 1. Introduction

### Cholesterol Metabolism in the Brain

**Figure 1:**
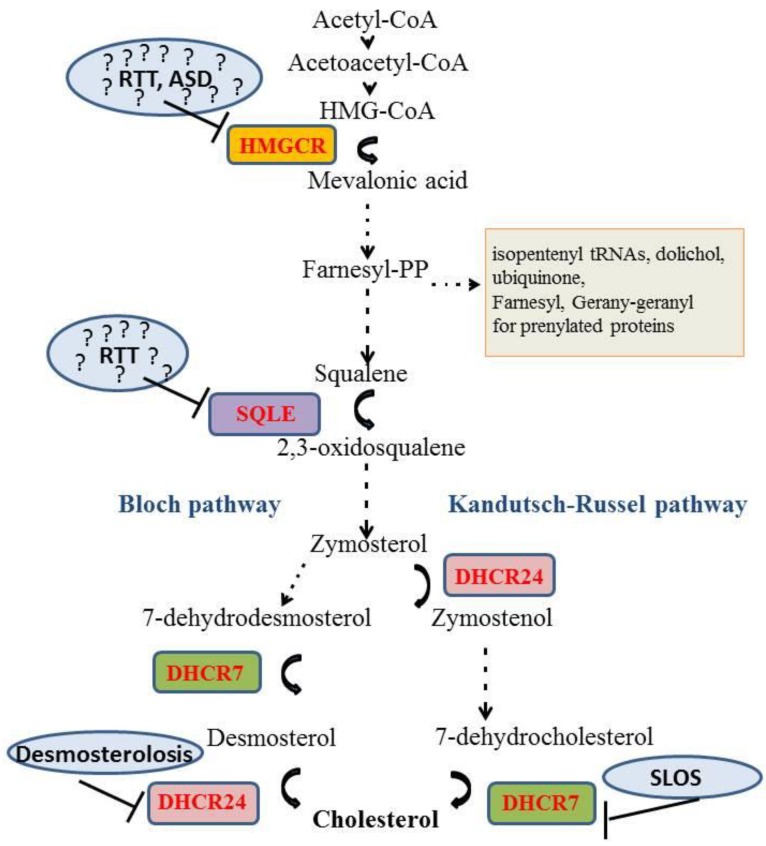
Schematic representation of MVA pathway. Enzymes involved or potentially involved in neurologic and psychiatric diseases are highlighted in blue circles. Autism spectrum disorder (ASD), 3-hydroxy-3-methylglutaryl Coenzyme A reductase (HMGCR); 7-dehydrocholesterol reductase (DHCR7); 24-dehydrocholesterol reductase (DHCR24), Rett syndrome (RTT); Squalene Epoxidase (SQLE); Smith–Lemli–Opitz syndrome (SLOS). Dotted arrows represent multiple enzymatic reactions. ? represents the hypothetic involvement of the indicated enzyme in the onset of the disease.

The brain contains 23% of the cholesterol in the human body although it represents only the 2% of the total body weight [1] indicating the importance of this molecule for the central nervous system (CNS). Cholesterol is a key constituent of myelin sheaths and neuronal membranes [2]. Specifically, cholesterol is a main component of synaptic vesicles: their formation, shape, and release are critically dependent on this lipid. Moreover cholesterol plays a role in the proper structural organization of postsynaptic densities [3]. Cholesterol is not homogeneously dispersed within membranes: it is more abundant in the cytosolic part of the plasma membrane [4] and it is enriched in dynamic structures called “lipid rafts” that play important roles in signal transduction across membranes [5]. In neurons, lipid rafts have been observed at synapses, where they could contribute to pre- and postsynaptic function [6,7,8,9,10]. Thus, an imbalance in cholesterol homeostasis both at the presynaptic and postsynaptic side is likely to impact neurotransmission, and induce the loss of synapses and dendritic spines [11,12]. Remarkably, cholesterol metabolism in the brain is completely separated from the rest of the body since lipoproteins containing cholesterol cannot cross the blood brain barrier (BBB). Cholesterol present in the CNS must be synthesized in situ [13]. The synthesis of this lipid is assured by the so-called mevalonate (MVA) pathway [14] that starts from acetate and comprises a series of ~30 enzymatic reactions. A key enzyme of this pathway is the 3-hydroxy-3-methylglutaryl Coenzyme A reductase (HMGCR), an integral membrane protein in the endoplasmic reticulum (ER) that reduces the 3-hydroxy-3-methylglutaryl Coenzyme A (HMG-CoA) to mevalonic acid (MVA). The membrane embedded portion of HMGCR contains a sterol-sensing domain (SSD) with five helices that is shared by several proteins involved in cholesterol homeostasis [15]. The SSD of HMGCR is crucial for the regulated degradation of the enzyme by the proteasome [16]. Importantly, MVA is the precursor of compounds other than cholesterol that are involved in diverse cellular processes such as transcription (isopentenyl tRNAs), protein *N*-glycosylation (dolichol phosphate), protein prenylation (farnesylation and geranylgeranylation), and mitochondrial electron transport (ubiquinone) (Figure 1) [17].

Several studies suggest that neurons produce cholesterol during prenatal life and depend on cholesterol synthesis by astrocytes after birth [12,13]. In the mouse CNS, cholesterol is synthesized at a rate of 0.26 mg/day during the first week of life. In adult animals, the synthesis exceeds the need and surplus cholesterol is excreted to plasma a rate of about 0.023 mg/day [18]. To this end, cholesterol is transformed by cholesterol 24-hydroxylase (CYP46A1) into 24-*S* hydroxycholesterol (24-*S*-OH), which is able to cross the BBB. The 24-*S*-OH is continually produced in the brain, mainly by neurons, and excreted into the bloodstream both in rodents and in humans [18]. This seemed to be the main mechanism to remove cholesterol from CNS [19,20], but data obtained by gas chromatography (GC)-MS have shown that the cholesterol metabolites 5α-hydroxy-6-oxocholesterol (3β,5α-dihydroxycholestan-6-one), 7β-hydroxycholesterol and 7-oxocholesterol, generally considered to be formed through reactive oxygen species, are similarly exported from brain at rates of about 0.1, 2 and 2 mg/24 h, respectively [21]. Brain cholesterol metabolism, in particular the MVA pathway, display region-, age-, and sex-specific differences. It has been shown that brain regions differ with respect to the activity and the content of proteins mediating cholesterol homeostasis. In particular, our research group demonstrated that cholesterol biosynthesis and uptake are differently modulated in different brain regions of adult male rats. The HMGCR activity is very low in the brain stem, and high in hippocampus, brain cortex and cerebellum. These regional differences depend on sex- and age-specific metabolic regulation [22,23].

## 2. Diseases with Direct Links to Cholesterol Homeostasis

The eminent importance of the cholesterol/MVA pathway for brain development and function is illustrated by hereditary albeit rare diseases that affect genes mediating cholesterol homeostasis (Table 1).

The autosomal recessive Smith–Lemli–Opitz syndrome (SLOS, OMIM 270400) is caused by mutations in the gene encoding 7-dehydrocholesterol reductase (DHCR7). This enzyme catalyzes the last step in the Kandutsch–Russell branch of the cholesterol/MVA synthesis (Figure 1). The most severe mutations of this gene cause fetal or newborn death, while less severe variants cause developmental defects in specific organs such as facial and cranial malformations, hypospadia or complete gonadal absence [24]. Some SLOS patients show intellectual disability, perturbed sleep, delayed acquisition of motor and language skills as well as impaired social interactions. The mechanisms causing this range of symptoms are not well understood. Evidently, dysfunction of DHCR7 will lead to lower cholesterol levels. In fact, the most severely affected patients present low cholesterol content down to 2% of the normal levels [25]. Low cholesterol levels may impair sonic hedgehog signaling and cause the striking developmental malformations. Activity of this signaling factor and its distribution are regulated by posttranslational attachment of cholesterol to its amino terminus [26,27,28].

SLOS patients with neurologic symptoms can present normal amount of plasma cholesterol probably due to dietary intake. Nevertheless, diet-derived cholesterol cannot cross the BBB and cannot compensate the low cholesterol level in the brain [3,24]. It is also possible that neurologic symptoms are caused by accumulation of 7-dehydrocholesterol (7-DHC), a DHCR7 substrate, or its oxidized metabolites [29]. It has been demonstrated that the accumulation of 7-DHC in fibroblasts impairs intracellular cholesterol transport and increases the degradation rate of HMGCR, contributing to a reduced sterol synthesis in SLOS patients [30]. The therapeutic goal for the treatment of SLOS should be to increase cholesterol content and to decrease the accumulation of the cholesterol precursors such as 7-DHC. Cholesterol supplementation in SLOS patients showed unclear results. Some reports described an improvement in alertness, attention, mood and affection, whereas others demonstrated no ability to restore any developmental acquired skills [31,32,33]. Potentially toxic cholesterol precursors may be lowered by treatment with HMGCR inhibitors, the statins, although this treatment may appear counterintuitive and paradoxal for syndrome caused by defect in cholesterol synthesis. Treatment of SLOS subjects with simvastatin and cholesterol demonstrated irritability and self-injury, improved appetite and sleeping patterns, while no change was detectable in the IQ score [34]. A recent study showed that treatment of a SLOS preclinical rodent model with cholesterol plus suitable antioxidants completely prevented the retinal degeneration [35] providing some hopes to SLOS patients.

A second genetic disorder that is directly linked to cholesterol is Niemann-Pick type C disease (NPC, OMIM 257220). The pathology is characterized by intracellular accumulation of unesterified cholesterol and glycolipids in the endosomal/lysosomal system [36]. Approximately 95% and 5% of cases are caused by mutations in the ubiquitously expressed Niemann-Pick type C 1 (NPC1) and NPC2 gene, which encode for a large membrane glycoprotein in late endosomes, and for a small soluble lysosomal protein, respectively. Structural analyses indicate that both molecules cooperatively mediate the exit of unesterified cholesterol from the endosomal/lysosomal system. NPC patients present with highly variable age of onset, neurovisceral symptoms, and life-span ranging from a few years to decades of life. A hallmark present in nearly all patients is supranuclear gaze palsy, other clinical manifestations are cerebellar ataxia, movement disorders, epileptic seizure, dysartria, cataplexy, dysphagia, vertical supranuclear ophthalmoplegia, dystonia, and progressive memory deficits as well as dementia [37]. Interestingly, patients with the same mutation show very different pathological symptoms [36,38]. The large symptomatic spectrum suggests the presence of disease modifiers that are still largely unknown. At present, there is no curative treatment for this disease, and reduction of glycolipids by *N*-butyl-deoxynojirimycin (Miglustat) is the only therapeutic option in the EU [39]. Recent data suggest a new therapeutic drug candidate, β-cyclodextrin [40], which may resolubilize cholesterol within cells and induce the release of cholesterol-rich membrane inclusions [41].

A third autosomal recessive disease with direct links to cholesterol is desmosterolosis (OMIM 602398), which is caused by mutations in the 24-dehydrocholesterol reductase (DHCR24) (Figure 1). This enzyme catalyzes the reduction of desmosterol to cholesterol, a crucial step in the Bloch branch of cholesterol synthesis (Figure 1) [42]. Patients with desmosterolosis present microcephalia, hydrocephalia, ventricular enlargement, defects in the corpus callosum, and thinning of white matter and seizures. As for SLOS, the symptoms of this disease may be attributed to reduced cholesterol levels or to accumulation of a precursor, in this case desmosterol. There is currently no therapy for this disease.

## 3. Neurodegenerative Diseases with Suspected Links to Cholesterol Metabolism

Several diseases including Alzheimer, Huntington, and Parkinson diseases are thought to involve changes in cholesterol metabolism although a direct link cannot be established [14] (Table 1). Alzheimer’s disease (AD, OMIM 104300) is a neurodegenerative disorder characterized by progressive behavioral and mood alterations including memory loss. AD is considered a proteopathy due to the intracellular and extracellular accumulation of neurofibrillary tangles made of tau and of plaques containing amyloid beta, respectively in the CNS of patients. Cholesterol has been implied in the production and clearance of beta amyloid [24]. Moreover, several proteins involved in cholesterol homeostasis have been associated with AD. A specific variant of the gene encoding for apolipoprotein E, which transports cholesterol in the brain, is one of the best established risk factors for late onset/sporadic AD [43]. Two polymorphisms of *CYP46* have been related to AD [44,45]. Finally, AD-related polymorphisms were detected for ATP binding cassette 1 protein (ABCA1), a protein that transfers cholesterol from cells to apolipoprotein A1-containing lipoproteins [46]. Several clinical trials using statins have been started, for the moment, the results seem to be debated [47]. Recently, however, a direct interaction between statins and amyloid beta has been demonstrated in silico [48].

Huntington’s disease (HD, OMIM 143100) is an autosomal-dominant disorder characterized by adult onset, progressive motor dysfunction, dementia, cognitive decline and psychiatric disturbances, which leads to death, approximately 15–20 years after disease onset. CAG repeats in the gene encoding for the huntingtin protein (HTT) are the cause of this disease. HTT plays a role in vesicle transport and cytoskeletal anchoring in clathrin-mediated endocytosis, neuronal transport and postsynaptic signaling; also, it protects neuronal cells from apoptotic stress and therefore may have a central function in cell survival [49]. However, it remains unclear whether and how mutant huntingtin causes neuronal degeneration and death. It has been demonstrated in vitro that mutant HTT represses genes involved in cholesterol metabolism [50] possibly due to a lower transcriptional activity of the Sterol Regulatory Element Binding Protein 2 (SREBP2). This transcription factor regulates a cassette of genes involved in cholesterol homeostasis [3]. Somewhat conflicting findings in HD mouse models gave rise to contrasting hypothesis regarding the HD-dependent cholesterol alteration: the first suggests that neurons suffer from a lack of cholesterol due to impaired biosynthesis [51,52]. The alternative hypothesis assumes an accumulation of cholesterol in membranes causing a decreased synthesis and a paralleled reduction in the production of 24-*S*-OH by CYP46A [20]. Accordingly, restoring normal levels of cholesterol is preclinically explored using administration of cholesterol-laden nanoparticles [53] and the increase of CYP46A levels using gene therapy [20], respectively.

Several studies suggest possible correlations between mood disorders and altered cholesterol levels, both in brain and blood [82]. Statins induce side effects such as irritability and violence [82,83] and a case study showed complete reversal of these changes upon suspension of drug treatment [84]. Other studies suggest a correlation between cholesterolemia and psychiatric alterations such as suicide [85,86,87] and depression, the latter of which may be sex-dependent [88,89,90]. An association between schizophrenia and plasma cholesterol level has been described since 1952 [90,91,92,93]. However, the studies correlating cholesterolemia and behavior have to be carefully considered since blood levels of cholesterol are completely independent from the brain and correlations cannot establish causal relations.

## 4. Autism Spectrum Disorder

A connection between autism spectrum disorder (ASD) and mevalonate/cholesterol metabolism has been suggested recently. ASD refers to a range of developmental psychiatric disorders characterized by deficits in social communication and interactions, restricted interests and repetitive behaviors that appear during the first two years of life [58]. 

In the fifth edition of the Diagnostic and Statistical Manual of Mental Disorders [59], the traditional three symptom domains of ASD (i.e., social impairment, communication deficit, and atypical/repetitive behaviors) have been reduced to two, by combining the social and communication symptoms into one single diagnostic criterion of social-communicative deficits. The deficit in the socio-communicative domain appear already at infancy: babies with ASD may show lack or weak response to the parents’ voice, tend to not use their voice to attract attention to themselves, to express emotions or establish contact, and may be unresponsive to social stimuli, avoiding interaction with others. As the children get older, withdrawal from social interactions, indifference to social activities and deficits in social communication become more evident, while the language appear highly impoverished [58,94]. The second symptom domain of ASD includes restricted, repetitive patterns of behaviors, interests or activities [59]. 

No specific drug treatment is currently available for ASD. In line with the multitude of symptoms displayed by ASD patients, it is not surprising that different etiological components are involved in the pathogenesis of the disease, including genetic and environmental factors [95]. The concordance rate, ranging from 60–95% in monozygotic twins to 2–6% in dizygotic twins, indicates that genetic factors play a key role. Indeed, several single gene mutations, polymorphisms and copy number variations have been associated with ASD. However, the fact that the concordance is less than 100% in monozygotic twins indicates that environmental and epigenetic factors are also involved [96]. Indeed, several prenatal and perinatal risk factors have been identified, including exposure to intrauterine infections, maternal treatment with drugs such as valproic acid and thalidomide, and exposure to toxicants such as organophosphate insecticides or heavy metals [96]. For this reason, ASD is by now considered as a multifactorial disease [95,96,97]. A recent study reveals age- and region-dependent alterations of proteins involved in cholesterol metabolism in the brains of a well-established rat model [54] based on prenatal exposure to valproic acid (VPA) [55]. This includes changes in the activation state of HMGCR and in the levels of several receptors mediating cholesterol uptake and release. Adolescent VPA-exposed rats showed a strong increase of membrane-attached geranylgeranylated RhoA in the cerebellum, a reduced level of the farnesylated Ras in the Nucleus Accumbens and a reduction of oligodendrocytes were observed [55]. Intriguingly, some of these changes were sex-dependent [56,57]. The sex-dependency is in agreement with the dimorphic onset of ASD, which is more frequent in boys than girls with a ratio of 3:1 [98], and with the differences in cholesterol homeostasis between human males and females [23,99]. A prominent sex-dependent change in the brains of VPA-exposed rats concerns LRP1 (LDLR related protein 1) [55,56]. Interestingly, a de novo variant in a canonical splice site of *LRP1* has recently been identified in ASD patients [60]. LRP1 is involved in several signaling pathways including cholesterol and lipid metabolism. Defects in this protein result in degeneration of dendritic spines and synapses and in neuroinflammation [100]. These data suggest that genetic variants of *LRP1* exert their role in psychiatric diseases impairing more than one pathway. Similarly, other lipoprotein receptors from the same family, such as LRP2 and LRP8, have been implicated in autism and psychosis [60,61]. Taken together, these findings strongly suggest that alterations of MVA pathway and cholesterol metabolism are implicated in the pathogenesis of ASD, altering the availability and distribution of cholesterol and isoprenoids, both necessary for the proper brain function.

## 5. Rett Syndrome

Rett syndrome (RTT, OMIM #312750) is another disease without a direct link to cholesterol metabolism. However, several recent studies suggest a dysregulation of cholesterol metabolism. RTT. 

RTT was first identified by the pediatric neurologist Andreas Rett, upon the observation of identical stereotypies in different female patients. The first examinations led the scientist to believe that symptomatology was primarily associated to metabolic defects: for this reason, this pathology was initially named cerebroatrophic hyperammonaemia in 1966 [62]. Subsequently, the continuous growth of knowledge allowed to better characterize the disorder, which was officially accepted by the whole scientific community after 17 years from its discovery [63]. RTT is a X-linked neurological disorder affecting only female with a prevalence of 1 out of 10,000 live births [64]. The first neuronal symptoms appear at the age of 6–18 months. Their state worsens progressively leading to loss of previously acquired speech and motor skills, and the occurrence of stereotypic hand movements, irregular breathing, difficulties in walking and seizures [65]. In 95% of the cases, RTT is caused by mutations in gene encoding for methyl CpG binding protein 2 (*MECP2*). This protein plays a key role in gene silencing through methylation-dependent remodeling of chromatin structure, and suppresses gene transcription through the association with several co-repressors [14]. MeCP2 is ubiquitously present. At present, it is unclear, why and how defects in this protein impair neuronal functions. Its level of expression is particularly high in neurons suggesting that it contributes to the establishment and/or maintenance of neuronal maturation and plasticity [66,67]. Besides mutations in *MECP2* gene, some individuals develop atypical forms of RTT, which are associated with mutations in X-linked cyclin-dependent kinase-like 5 (*CDKL5*; OMIM #300203) or Forkhead box G1 (*FOXG1*; OMIM #164874). Furthermore, several RTT patients still harbor undefined mutations [67]. Individuals affected by RTT display a decrease in brain volume and a concomitant reduction in head circumference [68,69], which are strongly associated to a smaller size of neurons and to an enhanced compaction of cells, particularly at the level of layers III and V of the cerebral cortex, substantia nigra, thalamus, cerebellum, basal ganglia, hippocampus and amygdala [70]. Disruptions at neuronal level also involve a reduction in dendritic arborization [101], and the dysfunction related to synapse physiology represent another hallmark of the disease [71,76]. It is becoming increasingly clear that RTT is deeply associated to important metabolic alterations [67]. For instance, it has been observed that metabolic hormones such as leptin and adiponectin are increased in plasma derived from RTT patients [72,73], and abnormal carbohydrate metabolism is also present in cerebrospinal fluid of RTT individuals [74]. In addition, recent evidence demonstrates that RTT physiopathology is associated to deregulations in cholesterol metabolism.

The first report linking RTT to cholesterol metabolism demonstrated enhanced levels of plasma total cholesterol, LDL (low density lipoprotein)-cholesterol and HDL (High density lipoprotein)-cholesterol in RTT patients, whereas no significant change was observed in the amount of total triglycerydes [102], suggesting that dysbalance in plasma lipid profile is restricted to cholesterol metabolism. In addition, the authors reported a strong reduction of scavenger receptor class B type 1 (SR-B1) expression in fibroblasts derived from RTT patients. This protein mediates the uptake of cholesteryl esters from HDL and LDL particles [102]. Few months after, an independent group showed significant dysregulated expression of genes involved in cholesterol biosynthesis, such as *Hmgcr* and Squalene Epoxidase (*Sqle*), in both brains and livers of *Mecp2*-null mice and a concurrent reduction of cholesterol precursors and a decrease of cholesterol biosynthesis in the brain [77,78,79]. Alterations were also observed in the amount of serum cholesterol derived from *Mecp2*-null mice, which was significantly increased [77]. A subsequent transcriptome/proteome analysis further corroborated these findings indicating perturbation of cholesterol homeostasis in the brain cortex of *Mecp2*-null mouse model of RTT [80]. Recent studies provide evidence for and against mevalonate/cholesterol synthesis pathway as valid therapeutic target. Lovastatin significantly improved systemic lipid profile, ameliorated motor behaviors and increased lifespan of *Mecp2*-null mice [77], whereas a second study performed on *Mecp2*-deficient mice with a different genetic background show no effect of lovastatin [81]. These results suggest that so far unknown modifiers impact the efficacy of lovastatin treatment on brain functions. Our studies support an involvement of cholesterol metabolism in RTT patients harboring mutations in *Mecp2* gene [14,75]. In agreement with other preclinical and clinical data [77,102], we observed a significant increase of total cholesterol and LDL-cholesterol plasma levels, and a decrease of SR-B1 protein expression in primary fibroblasts derived from RTT patients [75]. Importantly, we reported a dramatic reduction in the activity of HMGCR. This finding was intriguingly supported by the fact that cholesterol biosynthesis is reduced in the adult brains of a *Mecp2*-null mouse strain [77,78]. Together with the reduction in HMGCR activity, we highlighted a concurrent increase in the protein amount of HMGCR in RTT fibroblasts. Other studies indicated that *Hmgcr* transcripts are increased in the brains of 28-day-old, as well as in the livers of 3- and 8-weeks *Mecp2*-null mice [67,77]. A rise in LDLR expression was noted in RTT fibroblasts [75]. These changes suggest that low intracellular cholesterol content, as a consequence of HMGCR activity suppression in RTT fibroblasts, induces the classic feedback mechanism with activation of SREBP2 and the increase of its transcriptional targets such as LDLR (LDL receptor) and HMGCR [23,55,103,104,105,106,107]. Furthermore, LDLR increase is also promoted by the decrease in degradative events, as suggested by the fall in Proprotein Convertase Subtilisin/Kexin type 9 (PCSK9, a protein involved in LDLR degradation) plasma levels in RTT individuals [75]. The reduced MVA production could affect the other products of the biosynthetic pathway such as ubiquinone, dolichol and, most importantly prenylation. The putative aberrant content of these molecules could deeply affect cellular physiology, so further investigations are required to better understand their prospective involvement in this disease.

## 6. Conclusions and Outlook

The MVA/cholesterol pathway is crucial for CNS development and function and consequently, any dysfunction of this fundamental metabolic pathway is likely to provoke pathologic changes in the brain. Mutations in genes directly involved in MVA/cholesterol metabolism cause a range of diseases, many of which present neurologic and psychiatric symptoms (e.g. SLOS, NPC, desmosterolosis). This raises the question whether other diseases presenting similar symptoms are related albeit indirectly to the MVA/cholesterol pathway. 

Up to now, there is no direct proof that ASD and RTT are caused by defects in MVA/cholesterol pathway. However, the available data clearly demand new studies to investigate the state of MVA/cholesterol homeostasis in RTT and ASD. This includes more efforts to clarify the reliability of experimental models, and to delve deeper into the role of cholesterol metabolism in this neurological disorders. For instance, it would be interesting to assess whether disruption in cholesterol biosynthesis occurs in atypical RTT patients harboring mutations on genes different from *Mecp2*. The analysis of different cholesterol precursors and metabolites in the cerebrospinal fluid of RTT patients could be useful in order to provide a more direct estimation of cholesterol metabolism in the brain. Moreover, it is imperative to study in a more general manner whether and how ASD and RTT imply changes in lipid metabolism including fatty acids and phospholipids. Overall, there are first hints that MVA/cholesterol pathway are affected in diseases such as ASD and RTT, but further studies are necessary to address causal links, to identify the underlying mechanisms and the specific brain cell types involved, and finally, to determine whether MVA/cholesterol metabolism is a potential pharmacological targets to treat these devastating diseases.

## Figures and Tables

**Table 1:** Highlights of the direct and indirect involvement of MVA pathway in some brain diseases and the experimental models used. Smith–Lemli–Opitz syndrome (SLOS); NPC (Niemann-Pick type C); Alzheimer’s disease (AD); Huntington’s disease (HD); Autism spectrum disorder (ASD); Rett syndrome (RTT); 7-dehydrocholesterol reductase (*DHCR7*); 24-dehydrocholesterol reductase (*DHCR24*).

Disease	Direct/Indirect Involvement of MVA Pathway	Experimental Model	References
SLOS	Direct *DHCR7*	Human	[24,25,26,27,28,29,30,31,32,33,34]
		Rodents	[35]
NPC	Direct *NPC1* or/and *NPC2*	Human	[36,37,38,39]
		Rodents	[40,41]
Desmosterolosis	Direct *DHCR24*	Human	[42]
AD	Indirect	Rodents	[43]
		Human	[44,45,46,47]
		Silico	[48]
HD	Indirect	Striatal mouse cell line	[49,50]
		Rodents	[20,51,52,53]
ASD	Indirect	Rodents	[54,55,56,57]
		Human	[58,59,60,61]
RTT	Indirect	Human	[62,63,64,65,66,67,68,69,70,71,72,73,74]
		Primary human fibroblasts	[67,75]
		Rodents	[76,77,78,79,80,81]

## Abbreviations

7-DHC	7-dehydrocholesterol
24-S-OH	24-*S* hydroxycholestero
AD	Alzheimer’s disease
ASD	Autism Spectrum Disorder
ABCA1	ATP binding cassette 1 protein
CNS	Central Nervous system
CDKL5	Cyclin-dependent kinase-like 5
CYP46A1	Cholesterol 24-hydroxylase
DHCR7	17-Dehydrocholesterol reductase
ER	Endoplasmic reticulum
HDL	High Density Lipoprotein
HD	Huntington’s disease
HMG-CoA	3-hydroxy-3-methylglutaryl Coenzyme A
HMGCR	3-hydroxy-3-methylglutaryl Coenzyme A reductase
HTT	Huntingtin
LDL	Low density lipoprotein
LDLR	Low density lipoprotein receptor
LRP1	LDLR related protein
MeCP2	Methyl CpG binding protein 2
MVA	Mevalonate
NPC	Niemann-Pick type C
PCSK9	Proprotein Convertase Subtilisin/Kexin type 9
RTT	Rett syndrome
SLOS	Smith–Lemli–Opitz syndrome
SQLE	Squalene epoxidase
SR-B1	Scavenger Receptor class B type 1
SREBP2	Sterol Regulatory Element Binding Protein 2
VPA	Valproic acid
